# General practitioners’ management of mastitis in breastfeeding women: a mixed method study in Australia

**DOI:** 10.1186/s12875-024-02414-4

**Published:** 2024-05-10

**Authors:** Lisa H. Amir, Sharinne B. Crawford, Meabh Cullinane, Luke E. Grzeskowiak

**Affiliations:** 1 Judith Lumley Centre, School of Nursing and Midwifery, La Trobe University, Victoria, Australia; 2 Breastfeeding Service, Royal Women’s Hospital, Victoria, Australia; 3 SPHERE Centre for Research Excellence, Department of General Practice, Monash University, Victoria, Australia; 4 Department of Health Services Research, Peter MacCallum Cancer Centre, Melbourne, VIC Australia; 5 College of Medicine and Public Health, Flinders Health and Medical Research Institute, Flinders University, Adelaide, South Australia Australia; 6 South Australian Health and Medical Research Institute, Adelaide, South Australia Australia

**Keywords:** Mastitis, Breastfeeding, Maternal health, Antibiotics, Guidelines

## Abstract

**Background:**

Mastitis is a common reason new mothers visit their general practitioner (GP). In Australia, the *Therapeutic Guidelines: Antibiotic* provides practical advice to GPs managing a range of infections, including mastitis. It is not known if Australian GPs prescribe antibiotics and order investigations as recommended for the management of mastitis.

**Methods:**

A convergent mixed methods design integrated quantitative analysis of a general practice dataset with analysis of interviews with GPs. Using the large-scale primary care dataset, MedicineInsight, (2021–2022), antibiotics prescribed and investigations ordered for mastitis encounters were extracted. Mastitis encounters were identified by searching ‘Encounter reason’, ‘Test reason’ and ‘Prescription reason’ free text field for the term ‘mastitis’; ‘granulomatous mastitis’ was excluded. Clinical encounters for mastitis occurring within 14 days of a previous mastitis encounter were defined as belonging to the same treatment episode. Semi-structured interviews were conducted with 14 Australian GPs using Zoom or telephone in 2021–2022, and analysed thematically. The Pillar Integration Process was used to develop a joint display table; qualitative codes and themes were matched with the quantitative items to illustrate similarities/contrasts in findings.

**Results:**

During an encounter for mastitis, 3122 (91.7%) women received a prescription for an oral antibiotic; most commonly di/flucloxacillin ([59.4%]) or cefalexin (937 [27.5%]). Investigations recorded ultrasound in 303 (8.9%), blood tests (full blood examination [FBE]: 170 [5.0%]; C-reactive protein [CRP]: 71 [2.1%]; erythrocyte sedimentation rate [ESR]: 34 [1.0%]) and breast milk or nipple swab cultures in approximately 1% of encounters. Analysis using pillar integration showed consistency between quantitative and qualitative data regarding mastitis management. The following themes were identified:

- GPs support continued breastfeeding.

- Antibiotics are central to GPs' management.

- Antibiotics are mostly prescribed according to *Therapeutic Guidelines.*

- Analgesia is a gap in the *Therapeutic Guidelines.*

- Low use of breast milk culture.

**Conclusions:**

Prescribing antibiotics for mastitis remains central to Australian GPs’ management of mastitis. Interview data clarified that GPs were aware that antibiotics might not be needed in all cases of mastitis and that delayed prescribing was not uncommon. Overall, GPs followed principles of antibiotic stewardship, however there is a need to train GPs about when to consider ordering investigations.

**Supplementary Information:**

The online version contains supplementary material available at 10.1186/s12875-024-02414-4.

## Background

Mastitis is a painful breast infection that is a common experience for new mothers [1]. Around one in five breastfeeding women experience at least one episode of mastitis. While mastitis can occur at any stage of lactation the highest incidence is in the first four weeks after birth [2]. Women experiencing mastitis in the first month after birth are more likely to stop breastfeeding abruptly and to have stopped breastfeeding by six months compared with women not reporting mastitis [3]. New mothers experiencing mastitis are recommended to consult their doctor [4], yet little is known about how mastitis is managed by general practitioners (GPs) in Australia. Internationally, there is a paucity of research on how medical practitioners support breastfeeding women [5].

Mastitis is understood to be an inflammatory condition occurring along a spectrum from mild inflammation to bacterial infection to abscess development [6–8]. Mastitis is often understood to be synonymous with breast infection in the medical literature (“an infectious condition of the breast” ([9] p. 293). Women presenting to medical professionals tend to be at the more severe end of the spectrum with fever and established breast inflammation.

In Australia, many GPs use the *Therapeutic Guidelines: Antibiotic* to guide their management of conditions such as mastitis. The *Therapeutic Guidelines* state that it is safe for women with mastitis to continue breastfeeding and lists appropriate antibiotics [10]. Since at least 1998, flucloxacillin has been recommended as first choice. However, evidence from the UK suggests that it is not uncommon for inappropriate antibiotics (i.e., ones that the causative bacterial agents are likely to be resistant to) such as amoxicillin, to be prescribed [11, 12]. If women are prescribed the incorrect antibiotic they are likely to have a longer period of illness which may require admission to hospital, they may develop an abscess, or they may stop breastfeeding earlier than they planned [13].

While investigations are routine for many infections in general practice, for example, mid-stream urine testing for patients with suspected urinary tract infections, few investigations are conducted on the lactating breast. Mastitis is regarded as a “clinical diagnosis”, and milk culture is recommended only for patients with sepsis or who do not respond to first-line treatment [9]. Breast ultrasound is recommended when a fluctuant breast mass is present or if mastitis is not resolving and an abscess is suspected [9, 14].

Given the lack of research into the management of mastitis within the Australian context, this study had two broad aims: (1) to describe how GPs around Australia treat mastitis with antibiotics to determine if they are following best practice guidelines, such as the *Therapeutic Guidelines: Antibiotic* [10], and (2) to understand how GPs make decisions about prescribing for breastfeeding women, and how they use guidelines. To address the first aim, we analysed GP prescribing patterns for women with mastitis using the MedicineInsight dataset of Australian general practice electronic records. The second aim was addressed by conducting interviews with GPs to explore their management of mastitis and use of the *Therapeutic Guidelines.* In this paper we integrate data from the GP consultation dataset and individual interviews.

## Methods

### Philosophical approach

The quantitative component of this study involved an analysis of Australian GP practice data (i.e., entries from medical records of GPs) in the MedicineInsight dataset. This component fits the positivist paradigm with a logical deductive approach. Although this reflects an empiricist epistemology, we recognise that there are multiple flaws in these assumptions (e.g., have GPs diagnosed mastitis correctly? Did patients purchase the antibiotics and take them?). In contrast, the qualitative component is based on a more interpretivist approach, involving in-depth interviews with GPs. We based the interview guide around the COM-B (‘capability, opportunity, motivation-behaviour’) system as a framework for understanding the barriers/enablers to GPs’ use of guidelines [15]. We used an inductive approach to coding and analysis of the interviews. We recognise that our attitudes influence the way we collect and analyse the data, and our prior knowledge of the topic, findings from the dataset study, and other factors were all be brought into our research conclusions. This component has a relativist ontology (meanings are constructed subjectively) and subjectivist epistemology (researchers are part of the investigation) [16]. In this paper, we bring the two components together with a pragmatic approach: there are multiple perspectives of reality or worldviews.

### Study design

We used a convergent mixed methods design, where quantitative and qualitative data are collected and analysed separately over a similar period, and results are merged and compared [17]. In this study, quantitative analysis of a large dataset and qualitative data collection and analysis occurred over the same timeframe, with regular interaction between the researchers working on each component. The interview guide about GPs’ use of guidelines *built* on the preliminary findings from the dataset, and findings from both components were *merged* for analysis [17]. The findings are described in a *weaving approach* by presenting quantitative and qualitative findings topic by topic [17]. The data are brought together in a joint display [18], the Pillar Integration Process (described below) [19]. We followed the Good Reporting of A Mixed Methods Study (GRAMMS) framework for writing up the study [20].

### Quantitative component

MedicineInsight is a large-scale primary care dataset of longitudinal de-identified electronic health records (EHRs) in Australia [21]. The MedicineInsight program collates routinely collected EHR data from clinical information systems from consenting general practices; currently over 500 practices with over 3,000 GPs involved. It includes information from 9% of all Australian GPs and 13% of all Australian patients who saw a GP at least once during the financial year (2018–2019) [22].

The independent MedicineInsight Data Governance Committee approved the quantitative component (protocol 2019–003) and the Human Research Ethics Committee of the University of Adelaide and La Trobe University exempted it from ethical review due to the use of non-identifiable data.

Using data from 2021–2022, we restricted our analysis to females of reproductive age (18–44 years inclusive) with one or more documented clinical encounters related to mastitis and documentation relating to a pregnancy within the previous 12-months of the encounter. Mastitis encounters were identified by searching the ‘Encounter reason’ free text field for the term ‘mastitis’. We also searched the ‘Test reason’ and ‘Prescription reason’ free text field for the term ‘mastitis’. We excluded the free text term ‘granulomatous mastitis’ as this was considered unlikely to be related to lactational mastitis. Clinical encounters for mastitis occurring within 14 days of a previous mastitis encounter were defined as belonging to the same treatment episode. Only the first episode per individual was included in the analysis. Documented pregnancies were identified using the separate ‘pregnancy’ dataset which included data on date of last menstrual period and estimated date of confinement. We also searched the ‘Encounter reason’ free text field using terms related to pregnancy (i.e., ‘Antenatal’, ‘Pregnancy’, ‘Hyperemesis gravidarum’, ‘Morning sickness’), postpartum (‘postnatal’, ‘postpartum’, ‘baby check’, ‘6 week check’), or breast feeding (i.e., ‘breast feeding’, ‘breastfeeding’, ‘lactation’) to identify women with a recent pregnancy. This was undertaken to increase the likelihood of the clinical encounter being related to lactational mastitis. Notably, the MedicineInsight program uses the terms sex and gender interchangeably and presents sex/gender information as a single binary variable (i.e., female/male). Pensioner concession status is an indication of low income and was extracted as yes/no.

We report the proportion of women prescribed oral antibiotics on the same date as a mastitis encounter. Prescribed antibiotics were identified from the corresponding ‘Prescriptions’ dataset. Secondary outcomes included the proportion of women ordered clinical investigations for mastitis including breast ultrasound, breast milk culture, nipple swab culture, blood test (i.e., C-reactive protein [CRP], Erythrocyte Sedimentation Rate [ESR], Full Blood Examination [FBE]), and breast aspirate. These were identified by searching the ‘Requested tests’ free text field for the previously listed terms. Additional secondary outcomes included the proportion of women prescribed other medications, including topical or intravenous antibiotics, antifungals, lactation suppressants (i.e., cabergoline, bromocriptine), or lactation stimulants (i.e., domperidone). We are assuming “ultrasound” applies to a diagnostic ultrasound, but may also refer to therapeutic ultrasound, therefore we recognise the estimate for ultrasound is a likely to be an overestimate of number of actual diagnostic ultrasounds ordered. The dataset only includes biochemistry pathology results, so we were unable to analyse bacteriology or radiology data. Stata MP 17 (Stata, College Station, Texas) was used for analysis of the MedicineInsight dataset.

### Qualitative component

The qualitative component used semi-structured interviews to explore GPs’ perspectives of the issues they faced when managing mastitis, making decisions about prescribing medications, and how they used guidelines, such as the *Therapeutic Guidelines*. The qualitative component received approval from La Trobe University Human Research Ethics Committee (HREC Ethics Application Number: HEC21054). The study followed all relevant guidelines and regulations for conducting ethical research.

#### Recruitment and procedure

An invitation to participate in the study was posted on the Facebook group GPDU (GPs Down Under) with approval from the group administrator. The group has over 9,000 GP members from around Australia. The invitation briefly explained the purpose of the research and what participation involved, with a stock image (female doctor with a female patient) and a link to a short survey in REDCap [23, 24]. Interested GPs provided basic information to assess their eligibility (i.e. had seen breastfeeding woman in previous year; location; gender; age) and their contact details. The invitation was posted on 17 May 2021 and 27 October 2021. We received between 5 and 10 expressions of interest after each post. Several GPs were recruited using snowballing from initial participants. Eligible participants were contacted via email and sent the Participant Information and Informed Consent Form and an interview was arranged at a convenient time. They were asked to return the signed Informed Consent Form (via post or electronically) prior to the interview.

Interviews were conducted by MC and SBC between June 2021 and March 2022. The interviews were conducted online, using the Zoom platform, or via telephone. Each interview lasted between 30 to 45 min. The interviews were audio recorded, with permission, and the audio-recording was transcribed verbatim by a professional transcribing service and anonymised before analysis. Transcripts of interviews were emailed to participants to allow for member checking and verification prior to analysis.

Directly after each interview, the researcher made field notes of general impressions and reflections from the interview. After each interview, participants were sent an AUD$100 gift voucher to acknowledge their time commitment.

#### Interview schedule

A semi-structured interview schedule was used to guide the interviews. We based the Interview schedule on the Capability, Opportunity, Motivation-Behaviour (COM-B) framework which is structured to understand clinicians’ behaviour and likely barriers to perform according to best practice [15]. The schedule covered the following topics: *Capability* includes knowledge about prescribing during lactation, *Opportunity* includes social norms about management of breastfeeding women, and *Motivation* includes reflective aspects (beliefs around use of guidelines) and automatic aspects (established habits in prescribing for women with mastitis). The COM-B theory/framework has been useful in exploring barriers and enablers of Australian GPs’ management of children’s check-ups [25]. Basic participant demographic data were also included in the interview schedule, to help describe the sample. For example, participant gender, location, years’ experience as a GP, where they conducted their GP training and the number of children they had, were collected at the beginning of the interview. The schedule was adapted in an iterative manner and the final version is provided as a supplementary document (Additional file 1. Interview guide).

### Research team and reflexivity

LHA is an expert in mastitis and breastfeeding medicine research and led the study. LEG is an expert in pharmacoepidemiology in pregnancy and lactation and serves as an expert adviser to the *Therapeutic Guidelines*. He led the analysis of the MedicineInsight dataset for the quantitative study component. SBC has a background in health promotion and is an experienced mixed-methods public health researcher. She conducted interviews and led the analysis of the qualitative component of the study. MC has a background in microbiology and over ten years’ experience in breastfeeding/early parenting research. She arranged and conducted interviews. Prior to the interviews, SBC and MC conducted practice interviews with LHA who role-played different GPs to familiarise the interviewers with the topic, and allowed for minor changes to the interview schedule. Regular meetings helped to provide different perspectives on the findings during data collection and preliminary analysis phases, with LHA providing an insider view as a medical practitioner and the other team members reflecting on their experience as parents of young children, and experience from other research projects.

### Data analysis

#### Quantitative component

We tabulated the following:

- Proportion of women presenting with mastitis who are prescribed antibiotics, and antibiotic class;
- Investigations ordered during consultation: blood tests (FBE, CRE, ESR), ultrasound, breast milk and nipple swab culture.

#### Qualitative component

SBC led analysis of the interviews, using a thematic analysis approach based on Green and colleagues’ four stage coding framework (data immersion, coding, creating categories, and identifying themes) to identify key themes and issues around GPs’ decision making around the management of mastitis in breastfeeding women and their use of guidelines for mastitis [26, 27]. NVivo software was used to store and manage the data and support data analysis (QSR International). An iterative process to data analysis was used. Initially, SBC and LHA independently coded the first five interviews inductively, developed codes and compared coding. The codebook was then revised after discussion and consensus with team members. SBC coded the remaining interviews independently. LHA listened to recordings of all interviews while confirming written transcripts. Team members SBC, LHA and MC continued to meet to discuss the coding structure and reflect on the interviews, and the codebook continued to be refined until a consensus was reached. Data collection continued until a wide range of participants had been included and the team considered that no new codes were appearing (data saturation). Themes were generated based on the codebook, in collaboration with the team.

#### Integration

The research team met regularly to discuss the findings from both project components so the analysis benefitted from the knowledge acquired from both sources, and expertise from all members of the team.

We used the Pillar Integration Process (PIP) to develop a joint display table showing both quantitative and qualitative data [19]. The first step was to list the quantitative data on the outer left of the table, and summarise results as categories in the next column. Then, qualitative codes were placed in the outer right-hand column where they matched the quantitative items, with themes/categories in the next column. This process displays similarities or contrasts between the findings of the two components. Since our interviews covered a broader field than the quantitative data available in the GP dataset, we only used qualitative codes that relate to the management of mastitis (other qualitative data will be published separately). Where we had a qualitative theme that related to management of mastitis, but no relevant quantitative data were available in the dataset, we included the theme with an explanation, e.g., analgesia is important clinically. The team worked together to check the data were accurate and matching was complete and appropriate, and then patterns and insights were built in the Pillar column [19]. A separate paper will describe the barriers and enablers of GPs’ use of guidelines analysing the interview data using the COM-B framework. In addition, we prepared a report for the *Therapeutic Guidelines Ltd* and a poster depicting a GP consultation for a woman presenting with mastitis [28].

## Results

### Quantitative data

**Fig. 1:**
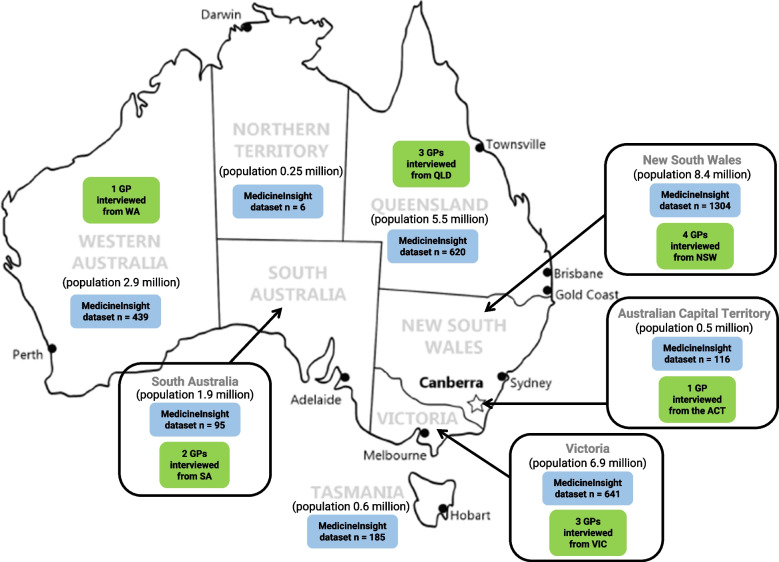
Distribution of participants in quantitative and qualitative components

Over 3,000 women with at least one episode of mastitis in 2021–2022 were identified in the dataset (*n* = 3046). Table 1 shows maternal age at encounter, concession card status, state or territory, and other characteristics of the sample. Most women were in their 30s, 13% had a Pensioner Concession Card, 69% lived in a major city and 2.5% were identified as Aboriginal and/or Torres Strait Islander. All states and territories were included, with 38% of the sample in NSW and 19% in Victoria. Figure 1 shows the geographical distribution of the sample as well as population in each State and Territory.

**Table 1:** Characteristics of women with mastitis in MedicineInsight dataset 2021–22 (*n* = 3046)

Characteristics	n (%)
**Age at first mastitis encounter**	
18–24	203 (6.0%)
25–29	714 (21.0%)
30–34	1413 (41.5%)
35–39	872 (25.6%)
40–44	204 (6.0%)
**Concession status**	
No Concession Card (Pension/DVA)	2963 (87.0%)
Pensioner Concession Card	443 (13.0%)
**Smoking status**	
Smoker	84 (2.5%)
Ex-smoker	985 (28.9%)
Non smoker	2034 (59.7%)
Not recorded	303 (8.9%)
**Remoteness**	
Major city	2338 (68.6%)
Inner regional	1037 (30.4%)
Remote & very remote	24 (0.7%)
Not recorded	7 (0.2%)
**Socioeconomic Status**	
Very low	369 (10.8%)
Low	598 (17.6%)
Middle	744 (21.8%)
High	799 (23.5%)
Very high	889 (26.1%)
Not recorded	7 (0.2%)
**Indigenous status**	
Aboriginal and/or TSI	86 (2.5%)
Neither Aboriginal nor TSI	3320 (97.5%)
**STATE**	
ACT (Australian Capital Territory)	116 (3.4%)
NSW (New South Wales)	1304 (38.3%)
NT (Northern Territory)	6 (0.2%)
QLD (Queensland)	620 (18.2%)
SA (South Australia)	95 (2.8%)
TAS (Tasmania)	185 (5.4%)
VIC (Victoria)	641 (18.8%)
WA (Western Australia)	439 (12.9%)

*TSI* Torres Strait Islander

### Qualitative data

We recruited 14 GPs from a range of settings in Australia. Most States and the Australian Capital Territory were represented (see Fig. 1), with most participants residing in an Australian capital city (*n* = 9). Four GPs reported working in regional or remote locations. Participants were mostly female, but included four male GPs; practice locations were 9 urban, 3 regional, and 1 remote. One GP, who practiced in an Australian capital city, also spent one week each month practicing in an outer regional location (see Table 2). When quoting participants, we use a number to identify them (P01 to P14).

**Table 2:** Demographic characteristics of general practitioners who took part in an interview

Characteristics	n (%)
**State/ Territory of residence** (*n* = 14)	
ACT (Australian Capital Territory)	1 (7)
NSW (New South Wales)	4 (29)
QLD (Queensland)	3 (21)
SA (South Australia)	2 (14)
VIC (Victoria)	3 (21)
WA (Western Australia)	1 (7)
^a^**Usual place of work** (*n* = 14)	
Major city in Australia	10 (71)
Inner regional Australia	1 (7)
Outer regional Australia	2 (14)
Remote Australia	1 (7)
**Aged 35 years or younger** (*n* = 13)	7 (54)
**Female** (*n* = 14)	10 (71)

^a^One General Practitioner working in a major Australian city indicated that they also practice in an outer regional town for one week each month

### Joint display integrating quantitative and qualitative data

Using the pillar integration process described above, we developed a joint display table integrating analysis of quantitative and qualitative data (Additional file 2). Data extracted from MedicineInsight dataset are presented in left hand columns, sorted by medications, investigations, and advice. Data from coding the GP interviews were matched in right hand columns, and quotes selected to illustrate the relevant codes. Where the dataset provided no data (e.g., analgesia is usually not included in prescribing data), no data are shown in the left-hand column, and the reason explained. The quantitative and qualitative data were added in an iterative manner and compared to look for agreement or discordance, and areas of missing data. The team discussed the analyses from both data sources and developed a theme to integrate the findings in each row, shown in the central column – “pillar integration”. Footnotes to Additional file 2 summarise the recommendations in the Therapeutic Guidelines [10].

We found a high agreement between the quantitative and qualitative data. The findings are organised according to the five pillar themes: Antibiotics central to GPs’ management; Antibiotic selection mostly appropriate; Investigations uncommon; Support to continue breastfeeding; and Analgesia may be underutilised.

### Antibiotics central to GPs’ management

Antibiotics were prescribed in over 90% of mastitis consultations: 3122 (91.7%) women received a prescription for an oral antibiotic (Additional file 2). In all GP interviews, the participants discussed their considerations for using antibiotics, when to start, which antibiotic to prescribe and issues involved in prescribing for breastfeeding women. Some participants described advising the patient to start the prescribed antibiotics if symptoms were not improving (“delayed prescribing”) [29]. However, from the quantitative data we are unable to measure the actual proportion of prescriptions dispensed by pharmacies or actually taken by patients.

*I would more often than not provide a script for antibiotics. And you've got choices; you can either start straight away, or you can do delayed scripts. So, education that they can wait 24 hours and then if things haven't improved, to start the antibiotics. (P12)*

Some participants mentioned the need to start antibiotics promptly. Here one participant describes possible consequences of not prescribing “early enough”:

*If you don’t prescribe it early enough, well I guess my concern is that it will develop into a full-blown abscess, or develop sepsis for the mum, and I think that would be a horrible consequence. So, it’s always better to respond earlier, I think. (P04)*

When asked about when antibiotics are needed, some participants explained that symptoms may have been present for over 24 h:

*First of all, how is she presenting? Does she have systemic symptoms? How long has she had symptoms for? So, if – I guess, some women do come in two hours after they have a symptom, you know? And if she’s just got a symptom, there’s no systemic symptom, she’s completely well, I’m happy for her to continue doing the non-antibiotic measures, for up to two days. But I would give her the script then and there, so that if it’s either not settling at two days, or if it’s starting to get worse, I’d tell her to fill the script. But usually, it’s already been going on for two days by the time they come in. And usually, they – because I work in this well-educated area – usually they’ve done those basic measures for two days and it’s not getting better. (P10)*

### Antibiotic selection mostly appropriate

Prescribing data in the MedicineInsight dataset showed that dicloxacillin or flucloxacillin were prescribed in over half of mastitis consultations (59%) (Additional file 2). The next most common antibiotic prescribed was cefalexin (28%). The *Therapeutic Guidelines* recommend di/flucloxacillin as first line treatment for mastitis as these are narrow spectrum antibiotics appropriate for the most common bacterial pathogen, *Staphylococcus aureus* [10]. Cefalexin is recommended for people with a penicillin allergy, unless the allergy is severe, in which case clindamycin is recommended [10]. Most participants were familiar with the *Therapeutic Guidelines* recommendations, and some mentioned using the online guidelines provided by the Royal Women’s Hospital, Melbourne, which includes a table showing recommended antibiotic regimens and potential side-effects [30]. However, some participants preferred to commence with cefalexin as they felt it was more convenient (see quote by P11 in Additional file 2) or more appropriate for community infections:

*Well look, I know that in terms of the advice is usually to do something like dicloxacillin or flucloxacillin for mastitis, I will always look at the severity first. At this very sort of early stage, very limited to a little area, then I will probably start more first line with Keflex [cefalexin], because I’m more comfortable with that in a community basis*. (P04)

While prescribing cefalexin which is a broad spectrum antibiotic (i.e. active against Gram negative and Gram positive bacteria) for reasons such as “convenience” or “comfort” is not in line with Australian guidelines [10], it may be appropriate in cases where the cause of postpartum fever is unclear and differential diagnoses include endometritis [31, 32].

### Investigations uncommon

The most common investigation ordered for women with mastitis was a diagnostic breast ultrasound in 9% of encounters; other investigations recorded were FBE in 5%, CRP in 2%, and ESR in 1%, breast milk or nipple swab cultures in approximately 2% (Additional file 2). ESR and CRP are non-specific inflammatory markers; CRP may be used to decide on antibiotic use, e.g., for respiratory infections [33].

In our interviews with GPs, we asked whether they ordered investigations when seeing women with mastitis, such as breast milk culture or breast ultrasound. Five participants said they had never ordered a breast milk culture and several said they asked patients to return for review if not improving – examples of both can be seen in Additional file 2.

It is concerning that a number of participants were unaware of the possibility of using a milk culture to confirm appropriate antibiotic selection: “*No, actually I haven’t done that before*” (P04).

Both our data sources indicated higher use of diagnostic ultrasound than microbiological testing; estimates from the dataset were 9% for ultrasound compared with about 2% for cultures. While one participant, P14, indicated frequent use (see quote in Additional file 2), most participants ordered a diagnostic ultrasound if breast lump persisted to rule out a breast abscess requiring drainage. Most participants were aware of the need for further evaluation if symptoms did not improve: “*If things don't improve, there's an abscess, then you go further, ultrasound*.” (P12). Our participants focused on not missing a breast abscess and some participants mentioned referring to a hospital or specialist if complications occur:

*. . . mastitis is this and if it’s not improving after treatment in 2-3 days get them back because it could be an abscess . . . If you don’t tell women about the red flags, they can end up with a breast abscess, they can become septic, women can die*. (P01)

*Yeah, fairly comfortable in managing it [mastitis], starting antibiotics and monitoring them, and then obviously having a threshold to be like when it's gone out of my comfort zone and you need to go to hospital, or it's turned into a breast abscess. I've had to send a few patients for that, so watching out for those sort of conditions*. (P06)

### Support to continue breastfeeding

Women experiencing mastitis may consider stopping breastfeeding because they feel so unwell [1], however sudden cessation increases the risk of abscess formation and continued breastfeeding is recommended [7]. The MedicineInsight dataset indicated very low prescribing of lactation suppressant medication (~ 1%). The galactagogue, domperidone, used to increase milk supply, was also prescribed in about 1% of consultations. While infant feeding advice was not recorded in the dataset, most of our interviewees reported that they encouraged ongoing breastfeeding, “*Continuing to breastfeed that’s very important*” (P14), and no-one mentioned stopping breastfeeding.

### Analgesia may be underutilised

In the row in the joint display table (Additional file 2) relating to analgesia, only qualitative data are shown because most analgesics used by women with mastitis are purchased over-the-counter and rarely prescribed and therefore not recorded in the GP dataset. Pain was commonly described as a presenting symptom of mastitis, but in response to our prompts about management of mastitis only five of our interviewees mentioned analgesia: “pain relief” (P01 and P09); “paracetamol” (P03); “for pain, ice packs, Panadol [paracetamol] and Nurofen [ibuprofen]” (P10); and “Nurofen [ibuprofen]” (P13). We coded these comments as “minimal use of analgesia” because most interviewed GPs did not mention any form or analgesia, and those who mentioned it did so briefly, almost dismissively.

## Discussion

We found consistency between the quantitative data in the MedicineInsight dataset and the qualitative interviews with GPs. Five themes were identified: Antibiotics central to GPs’ management; Antibiotic selection mostly appropriate; Investigations uncommon; Support to continue breastfeeding; and Analgesia may be underutilised.

### Antibiotics central to GPs’ management

The *Therapeutic Guidelines* promote the use of antibiotics and support delayed prescribing: “In patients with systemic symptoms, or symptoms or signs that have not resolved after 24 to 48 h of increased breastfeeding and expressing of milk, early antibiotic therapy is important to prevent abscess formation. Combine antibiotic therapy with increased breastfeeding and expressing of milk.” [10]. Advice to emergency physicians similarly urges “early antibiotic therapy... in all cases with symptoms greater than 24 h” ([9] p. 295]). In most situations, symptoms will be present for over 24 h by the time lactating women consult a GP, and therefore these recommendations are consistent with the high levels of antibiotic prescribing. However, the advice does recognise that not all cases of mastitis require antibiotics; general advice about relieving breast fullness, resting and applying cold may be all that is required [7].

A similarly high rate of antibiotic prescribing has been reported in a study of Croatian GPs: 93% reported prescribing an antibiotic [34]. A study from Taiwan investigating medical claims for postpartum mastitis in a national population-based database (2008–2017) identified that 79% of cases were prescribed antibiotics, mostly in the first month postpartum, as outpatients [35]. In the US, Foxman et al. reported 86% of women were prescribed antibiotics [36]. However, antibiotics are used to treat mastitis less frequently in Scandinavia, with only 15% of women receiving antibiotics in Kvist’s trial of acupuncture [37], 38% in Finland [38] and 37% in Norway [3].

### Antibiotic selection mostly appropriate

Foxman’s US-based study of over 900 women found that the most commonly prescribed antibiotics for mastitis were cephalexin (46%), amoxicillin (7%), ampicillin (7%), and amoxicillin and clavulanic acid (7%) [36]. While over one third of participants in the Norwegian Mother-Baby cohort did not know the name of the antibiotic they had taken (36.5%); the most commonly reported antibiotic was a penicillin 53.4% with 9.7% reporting a macrolide and only 1.6% reporting a cephalosporin [3].

### Investigations uncommon

Our finding of low rates of investigations is consistent with other studies: no cultures were performed by clinicians in Foxman et al.’s US study [36].

The advice in the *Therapeutic Guidelines* is “If infection does not resolve with antibiotic therapy, evaluate the patient for an abscess and consider whether infection is caused by another pathogen” [10]*.* Scott suggests monitoring and further evaluation if symptoms do not improve to rule out resistant bacteria, abscess or malignancy [7].

Other guidelines are more specific about when and how to conduct milk culture:

*A breastmilk culture is not necessary to guide antibiotic choice but may be useful in cases of treatment failure, antibiotic allergy, severe or frequent infections. If culture is needed, care should be taken to avoid skin contamination by first cleansing the nipple and areola with an alcohol swab and then expressing the milk into a sterile collection tube, such as those used for urine culture. *[8]* p. 526*

While routine milk culture is not needed, it is valuable in locations with high levels of methicillin-resistant *S. aureus* (MRSA) [7]. A recent hospital-based study in Milan, Italy found that 45% of *S. aureus* isolates in cases of mastitis/abscess referred to a Breastfeeding Unit (2016–2018) were MRSA [39]. In a study of women admitted to a hospital in China with mastitis or breast abscess, 35% of *S. aureus* isolates were MRSA [40]. In Ukraine, 28% of *S. aureus* isolated was MRSA in mastitis (18427 breastfeeding women who gave birth in 11 regional hospitals of Ukraine in 2015–2017) [41]. While MRSA is less common in Australia, estimates are low in Victoria and Tasmania, but high in the Northern Territory [42], and needs to be considered if there is a poor response to standard antibiotics [30]. Unusual presentations, such as bilateral mastitis or a cellulitic appearance may be associated with streptococcal infections [43].

### Support to continue breastfeeding

The safety of continuing to breastfeed during mastitis was strongly stated in the World Health Organization’s review of mastitis in 2000 [6], and reiterated in international guidelines since, including the *Therapeutic Guidelines* [7, 9, 10, 44]. We are not aware of any guidelines that recommend lactation suppression during an episode of mastitis.

In contrast to our findings, several studies have reported that doctors inappropriately advised cessation of breastfeeding. In Scotland, one in ten women (6/57) were inappropriately advised to either stop breastfeeding from the affected breast or to discontinue breastfeeding altogether [12]. A recent interview study in Israel found that women described a low level of knowledge among physicians’ about treating breastfeeding problems and some women with mastitis were given incorrect advice to stop breastfeeding because of the need to take antibiotics [45]. The survey of Croatian GPs found that 11% (12/110) recommended infant formula during mastitis and 5% (7/155) prescribed a prolactin suppressant [34].

### Analgesia may be underutilised

Analgesia was unable to be assessed using the MedicineInsight dataset, but was barely mentioned by our GP participants. Analgesia is also mentioned uncommonly in other studies of medical management: 35% in Croatia [34], 17% in the US [36].

In the section on management of mastitis in the *Therapeutic Guidelines*, analgesia is not mentioned [10]. Since analgesia is an important component of mastitis management, this is a gap in the *Therapeutic Guidelines* which needs to be addressed. This is a topic requiring research, as although non-steroidal anti-inflammatories are often recommended for mastitis (“NSAIDs also reduce mastitis-related inflammation and are compatible with breastfeeding” ([7] p. 74), efficacy trials comparing NSAIDS to paracetamol are lacking.

### Strengths and limitations

Our research design involved collecting and analysing quantitative and qualitative data concurrently, enabling a more complete understanding of current management of mastitis by Australian general practitioners than would be possible with a single method study. Typically, mixed methods studies present quantitative and qualitative data separately, whereas we set out to show the rigour of our mixed method approach by integrating the findings in a joint display table [46]. Additional file 2 shows that clearly framing the management of mastitis around the *Therapeutic Guidelines* was useful in comparing findings from the quantitative and qualitative data, and highlights the similarity between the data sources, as well as the gap in management exposed (i.e., the potential underutilisation of analgesia).

The limitations of using electronic general practice records is that we cannot be certain that all women were being treated for lactational mastitis. We made the assumption that prescriptions or clinical investigations ordered on the same day as a clinical encounter for mastitis were related to that encounter reason. However, they may have been provided for alternative indications. As mentioned above, providing a prescription does not automatically mean that patients purchase (and take) the prescribed medication. Alternatively, some patients may have pressured the GP to prescribe an antibiotic in a situation where it may not be warranted.

Our interviews included GPs with a range of experience, some familiar with breastfeeding problems, and others less familiar. However, since we don’t have observations from actual GP consultations, we can only report what participants said they did in practice. Participants recruited from a Facebook group may not be representative of all GPs. Our findings might be different if we had been able to record consultations, for example perhaps more emphasis is given to analgesia than is evident from our interviews.

## Conclusions

Our convergent mixed methods study of Australian GPs’ management of mastitis found congruency between the two data sources, a GP dataset and qualitative interviews. Prescribing antibiotics was central to GPs’ management in both the dataset analysis and interviews with GPs. Overall, GPs followed principles of antibiotic stewardship, however there is a need to inform GPs about when to consider ordering investigations as breast milk cultures may be underutilised. Australian GPs appear to provide support for continued breastfeeding during mastitis. GPs’ advice about analgesia for women with mastitis is unclear from this study, although they undoubtedly recognised that fever and pain are prominent symptoms. We recommend guidelines for clinicians strengthen their recommendations about the importance of analgesia for patients with inflammatory symptoms.

### Supplementary Information

**Additional file 1.** Interview guide.

**Additional file 2.** Summary of joint display table.

## Data Availability

*Quantitative component*: Data may be obtained from MedicineInsight and are not publicly available. Third parties may express an interest in the information collected through MedicineInsight. The provision of information in these instances undergoes a formal approval process and is guided by the MedicineInsight independent external Data Governance Committee. This Committee includes general practitioners, consumer advocates, privacy experts and researchers. *Qualitative component*: The dataset used during the current study is not publicly available due to privacy conditions set by the University HREC but are available from the corresponding author on reasonable request.

## Abbreviations

CRP: C-Reactive Protein
ESR: Erythrocyte Sedimentation Rate
FBE: Full Blood Examination
GP: General practitioner
MRSA: Methicillin-resistant *S. aureus*
NSAIDs: Non-steroidal Anti-inflammatory Drugs
*S. aureus*: *Staphylococcus aureus*
