# Supplementary material for: General practitioners’ management of mastitis in breastfeeding women: a mixed method study in Australia
Source: BMC Prim Care. 2024 May 10;25:161. doi: 10.1186/s12875-024-02414-4 (PMC11083748; doi:10.1186/s12875-024-02414-4)
Supplement: Supplementary file 1 — Additional file 1. Interview guide. [file 12875_2024_2414_MOESM1_ESM.docx]

**Interview Schedule – General Practitioners**

**SEMI-STRUCTURED INTERVIEW SCHEDULE FOR GENERAL PRACTITIONERS**

**‘Understanding antibiotic prescribing for mastitis in breastfeeding women: a mixed method study’**

The interviewer will record in notes:

- Date of interview
- Time interview commences, time interview concludes
- Participant number
- Non-identifiable detailed notes for the purpose of analysis, to be stored securely at La Trobe University

This table is a guide to the main themes and topics to be covered in the interviews with General Practitioners.

| **Structure** | **Main Topics for Discussion** | **Questions** |
| --- | --- | --- |
| Getting Started | - Greet individual and confirm that they want to participate. - Introduce self and the research. - Explain purpose of the interview and how findings will be used. - Reassure there are no right or wrong answers, responses will be de-identified. - Reminder that the interview will be recorded. | - Today I would like to hear your views and experiences about caring for patients with mastitis and about your use of guidelines, such as the *Therapeutic Guidelines,* in making decisions about treatment options. - We hope to use the information gathered today, and in interviews with other GPs, to better understand how guidelines are used by GPs regarding mastitis care, and to improve the useability of the *Therapeutic Guidelines* for breastfeeding women. - A reminder that the interview will be audio/visually recorded. We will only use the audio recording for the analysis. If you start to feel uncomfortable during the interview or need to stop for a break, let me know. - All information you provide will be treated as confidential and stored securely at LTU. You will not be identified in any written report or papers generated by the study. |
| Current GP work | - Participants’ demographic characteristics. - Participants’ GP history. | Before we begin, we are collecting some information to describe our sample.   - I can see from the Expression of Interest form that you completed that you are located in [*location name*], is this correct? And where did you complete medical school? Do you have any children? - Can you tell me a bit about your clinic/work? Where is your clinic located? How long have you worked as a GP? Do you have any particular fields of interest? |
| Knowledge, beliefs and experience about mastitis | - General understanding of mastitis - Engagement with patients with mastitis - Patient consultation - Level of comfort in discussing and diagnosing mastitis - Level of confidence in treating patients with mastitis. | This study focusses on mastitis in breastfeeding women.   - What would you say are the classic features of mastitis? [Area of the breast is red, hard and tender, and woman has fever and/or systemic symptoms such as lethargy, muscle aching, rigors, etc] - Can you briefly tell me how often you would see a patient with mastitis [prompt: every week, month, year, rarely]? - How comfortable are you in looking after women with breastfeeding problems? With mastitis? [prompt: very comfortable > uncomfortable] - How confident are you in managing mastitis? - Thinking about a typical consultation with a woman with suspected mastitis:   - Can you describe how she would present?   - What sort of things would you ask her about?   - What else would the consultation involve?   [prompt: asking questions, physical examination, use resources]   - If you have made a diagnosis of mastitis, what are the next steps? [investigations ordered (milk culture or diagnostic ultrasound), antibiotic prescribing, allergy information, referral (therapeutic ultrasound, hospital, other practitioner)] - Can you describe if/how experience with diagnosing and treating mastitis has changed over your career/time as a GP? [prompt: milk culture analysis for MRSA or other bacteria] - How do your colleagues treat women with mastitis? |
| Knowledge and use of resources for mastitis | - Resources used | We are interested in the resources used for mastitis.   - Have you heard about any guidelines for the management of mastitis? If so, what are they? - Do you use any guidelines/diagnostic tools/resources during the consultation if mastitis is suspected? [prompt: *Therapeutic Guidelines*, Royal Women’s Hospital, Australian Medicines Handbook (AMH), Primary Health Network pathways, Google, ABA, other].   - When you used any guidelines in the past, what is it about these resources that you like? [prompt: trust the information, ease of access, recommended dosage, length of use].   - Anything you don’t like? [prompt: accessibility]   - How easy are they to access? - If you have a question about mastitis, where (else) would you go for advice or information? [prompt: other GP, maternity hospital, infectious disease physician, breast surgeon, online resources such as Academy of Breastfeeding Medicine website, other?].   - What is it about these resources that you like? [prompt: trust the information, ease of access] - How do you make decisions about when to start antibiotics? Which one to select? [don’t prompt re delayed prescribing, but be alert for it!] - Do you think there are any harms if mastitis isn’t managed appropriately? [prompts: abscess formation] - Do you think there are any harms of following (or not) guidelines for the management of mastitis? [prompts: inappropriate prescribing] |
| Prescribing for breastfeeding women |  | - Can you describe how you make decisions about prescribing medication for breastfeeding women. [prompt: what process is followed?]   - How comfortable are you prescribing medications for breastfeeding women?   - How confident do you feel in accessing resources about the safety of medications during lactation?   - What goes through your mind when you’re making a decision about prescribing for a breastfeeding woman? [prompts: milk supply, transfer to infant] - How did you learn about prescribing for breastfeeding women? [prompts: working in the field a long time, medical school] |
| Knowledge and use of Therapeutic Guidelines for mastitis | - Knowledge of TGs for mastitis - Use and usefulness of TGs for mastitis | Thinking specifically about the *Therapeutic Guidelines (TGs)* for mastitis.   - Are you aware of these guidelines for managing/treating mastitis? - What do you think the guideline says? [prompt: most likely causative agent (*Staphylococcus aureus*), appropriate antibiotic and dose to prescribe, infant feeding recommendations]   - What is your understanding of the recommended treatment? When should treatment be commenced?   - What is your understanding of the infant feeding recommendations? (continue breastfeeding or not) - Do you use the TGs for mastitis? If not, why not? - If you do use the TGs:   - Can you describe how you use the guidelines?   - How useful do you find the TGs for mastitis?   - What is it about the TGs that you like re advising for treatment for mastitis? (prompt: trust the information, ease of access)   - Is there anything that you don’t like? - Is there anything that you would like to see in the TGs for mastitis that is not currently available for GPs? - Do you use the TGs for other breastfeeding problems?   - If so, for which conditions?   - How useful do you find the TGs for these conditions? - What would make it easier to access guidelines, like the Therapeutic Guidelines? [prompt: electronic/print/other?] |
| Antibiotic stewardship | - Appropriate prescribing of antibiotics | We are interested in appropriate prescribing of antibiotics.   - What are your thoughts on antibiotic stewardship?   [prompt: we mean the appropriate prescribing of antibiotics]   - How does it work in practice? Can you give me an example? |
| Closing | - Other issues the participant would like to discuss? - Interviewer thank the parent for participating. | - Is there anything else you’d like to add about the TGs for mastitis or in general? - Is there anything else you’d like to add about your experience caring for patients with mastitis or breastfeeding women in general? [prompts: conflicting advice, inappropriate advice] - Thank you for your time, and for sharing your experiences. We have some Coles-Myer gift cards that we would like to send you in appreciation of your time. Where would you like me to post these to [*get postal address if appropriate*]. - You can contact me with further information or concerns. - Once the transcript it ready, I will email you a copy and you will have an opportunity to review the transcript for accuracy and make any changes or additions. Can I confirm that your email address is… [*confirm from Expression of Interest Form/ obtain appropriate email address*]. |
