# Supplementary material for: General practitioners’ management of mastitis in breastfeeding women: a mixed method study in Australia
Source: BMC Prim Care. 2024 May 10;25:161. doi: 10.1186/s12875-024-02414-4 (PMC11083748; doi:10.1186/s12875-024-02414-4)
Supplement: Supplementary file 2 — Additional file 2. Summary of joint display table. [file 12875_2024_2414_MOESM2_ESM.pptx]

## Slide 1
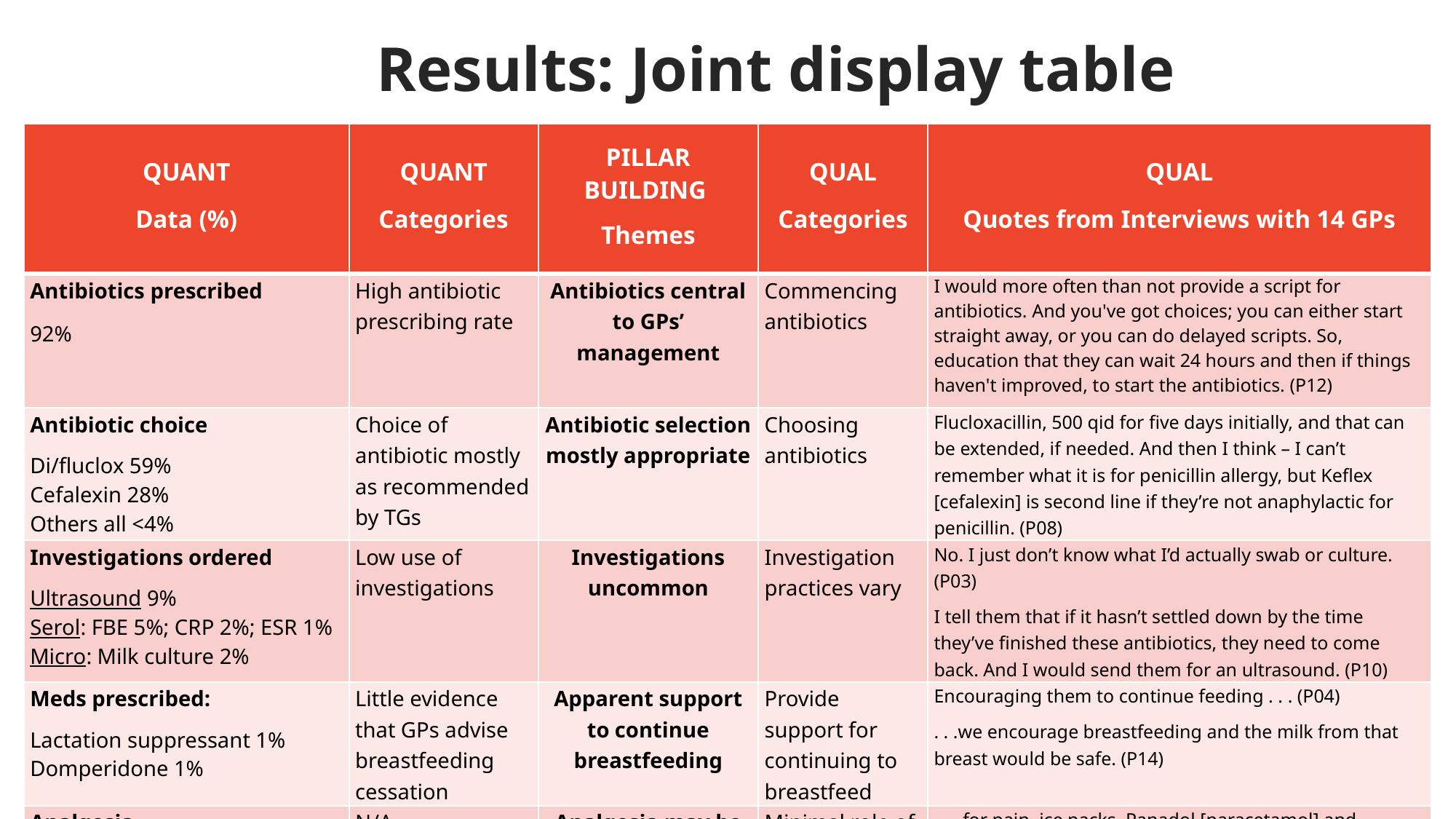

Results: Joint display table
| QUANT Data (%) | QUANT Categories | PILLAR BUILDING Themes | QUAL Categories | QUAL Quotes from Interviews with 14 GPs |
| --- | --- | --- | --- | --- |
| Antibiotics prescribed 92% | High antibiotic prescribing rate | Antibiotics central to GPs’ management | Commencing antibiotics | I would more often than not provide a script for antibiotics. And you've got choices; you can either start straight away, or you can do delayed scripts. So, education that they can wait 24 hours and then if things haven't improved, to start the antibiotics. (P12) |
| Antibiotic choice Di/fluclox 59% Cefalexin 28% Others all <4% | Choice of antibiotic mostly as recommended by TGs | Antibiotic selection mostly appropriate | Choosing antibiotics | Flucloxacillin, 500 qid for five days initially, and that can be extended, if needed. And then I think – I can’t remember what it is for penicillin allergy, but Keflex [cefalexin] is second line if they’re not anaphylactic for penicillin. (P08) |
| Investigations ordered Ultrasound 9% Serol: FBE 5%; CRP 2%; ESR 1% Micro: Milk culture 2% | Low use of investigations | Investigations uncommon | Investigation practices vary | No. I just don’t know what I’d actually swab or culture. (P03) I tell them that if it hasn’t settled down by the time they’ve finished these antibiotics, they need to come back. And I would send them for an ultrasound. (P10) |
| Meds prescribed: Lactation suppressant 1% Domperidone 1% | Little evidence that GPs advise breastfeeding cessation | Apparent support to continue breastfeeding | Provide support for continuing to breastfeed | Encouraging them to continue feeding . . . (P04) . . .we encourage breastfeeding and the milk from that breast would be safe. (P14) |
| Analgesia Usually OTC | N/A | Analgesia may be underutilised | Minimal role of analgesia | . . . for pain, ice packs, Panadol [paracetamol] and Nurofen [ibuprofen] (P10) |
